# Predicting the mechanism of phospholipidosis

**DOI:** 10.1186/1758-2946-4-2

**Published:** 2012-01-26

**Authors:** Robert Lowe, Hamse Y Mussa, Florian Nigsch, Robert C Glen, John BO Mitchell

**Affiliations:** 1 Unilever Centre for Molecular Sciences Informatics, Department of Chemistry, University of Cambridge, Lensfield Road, Cambridge CB2 1EW, UK; 2 Chemical Biology Informatics, Quantitative Biology, Developmental and Molecular Pathways, Novartis Institutes for BioMedical Research, 220 Massachusetts Avenue, 02139, Cambridge MA, USA; 3 Biomedical Sciences Research Complex and EaStCHEM School of Chemistry, Purdie Building, University of St Andrews, North Haugh, St Andrews, Scotland KY16 9ST, UK

## Abstract

The mechanism of phospholipidosis is still not well understood. Numerous different mechanisms have been proposed, varying from direct inhibition of the breakdown of phospholipids to the binding of a drug compound to the phospholipid, preventing breakdown. We have used a probabilistic method, the Parzen-Rosenblatt Window approach, to build a model from the ChEMBL dataset which can predict from a compound's structure both its primary pharmaceutical target and other targets with which it forms off-target, usually weaker, interactions. Using a small dataset of 182 phospholipidosis-inducing and non-inducing compounds, we predict their off-target activity against targets which could relate to phospholipidosis as a side-effect of a drug. We link these targets to specific mechanisms of inducing this lysosomal build-up of phospholipids in cells. Thus, we show that the induction of phospholipidosis is likely to occur by separate mechanisms when triggered by different cationic amphiphilic drugs. We find that both inhibition of phospholipase activity and enhanced cholesterol biosynthesis are likely to be important mechanisms. Furthermore, we provide evidence suggesting four specific protein targets. Sphingomyelin phosphodiesterase, phospholipase A2 and lysosomal phospholipase A1 are shown to be likely targets for the induction of phospholipidosis by inhibition of phospholipase activity, while lanosterol synthase is predicted to be associated with phospholipidosis being induced by enhanced cholesterol biosynthesis. This analysis provides the impetus for further experimental tests of these hypotheses.

## Background

Since the observation of phospholipidosis by Nelson and Fitzhugh in 1948 [1], many attempts have been made at understanding the underlying mechanism(s) [2,3]. Phospholipidosis is the excess accumulation of phospholipids induced in several cell types by numerous cationic amphiphilic drugs (CADs). The most reliable way of determining whether a compound has induced phospholipidosis is by electron microscopy. This analysis is important in the drug development process where the occurrence of phospholipidosis can cause delays and possibly termination of a project (as more tests need to be carried out to satisfy regulatory bodies). It is still unclear whether an accumulation of phospholipids is harmful to human health [4], the process is often reversible upon withdrawal of the compound, and despite attempts to understand the mechanism of phospholipidosis there is still no mechanistic understanding of how CADs can induce the accumulation of phospholipids in various cell types across different species.

A build-up of phospholipids can be explained by an inhibition of the breakdown or an increase in the synthesis of the phospholipids. Early studies supported the idea that inhibition of the breakdown of phospholipids was a possible mechanism. Hostetler *et al. *[2] showed strong support for the theory that the action of CADs was located in the lysosomes and that inhibition of the lysosomal phospholipases A and C caused a build-up of phospholipids. However, there was no way to distinguish between a drug-enzyme or drug-phospholipid binding event as the cause of the inhibition. Joshi *et al. *[5] tried to address this problem by measuring binding of phospholipidosis-inducing drugs to L-α-dipalmitoyl phosphatidylcholine vesicles. This suggested that if a drug was found to bind, then drug-phospholipid binding would be the cause of the inhibition of the phospholipases. While most of the drugs tested did bind to L-α-dipalmitoyl phosphatidylcholine vesicles, chloroquine (a phospholipidosis-inducing CAD) did not bind, suggesting that its main mechanism is the direct inhibition of one or more phospholipase enzymes. Abe *et al. *[6] produced the first study that distinguished between lysosomal phospholipases A1 and A2. This showed that two CADs, amiodarone and D-threo-1-phenyl-2-decanoylamino-3-morpholino-1-propanol, caused inhibition of lysosomal phospholipase A2. They found that no inhibition occurred on exposure to tetracycline, despite its being a CAD. Hirode *et al. *[7], however, found evidence that at high doses tetracycline may induce phospholipidosis. Further studies on lysosomal phospholipase A2 inhibition by CADs have been performed in which Hiraoka *et al. *[3] used lysosomal phospholipase A2 (LYPLA2)-deficient mice to study the relationship between LYPLA2 and phospholipidosis. A deficiency of the enzyme resulted in foam cell formation, surfactant lipid accumulation, splenomegaly (enlargement of the spleen), and phospholipidosis. A smaller number of studies have also looked at the possibility of increased synthesis of phospholipids being the mechanism for phospholipidosis by showing that an increase or redirection of synthesis leads to increased amounts of acidic phospholipids [8,9].

Reasor *et al. *[4] produced a review on the subject suggesting that no single underlying mechanism covers all phospholipidosis-inducing compounds. Phospholipidosis is not organ specific [10], however, it can be species specific where certain drugs cause phospholipidosis in one species and not in another. This implies that the mechanism for phospholipidosis may be highly complex and species dependent. Sawada *et al. *[11] recently summarised four possible mechanisms suggested by their toxicogenomics experiments:

1. Inhibition of lysosomal phospholipase activity;

2. Inhibition of lysosomal enzyme transport;

3. Enhanced phospholipid biosynthesis;

4. Enhanced cholesterol biosynthesis.

Attempts have been made to predict the occurrence of phospholipidosis using *in silico *methods. Ploemen *et al. *[12] suggested that a compound would be phospholipidosis-inducing (PPL+) provided that it has pK_a _> 8 and ClogP > 1 and that the sum of the squares (ClogP^2 ^+ pK_a_^2^) is greater than 90, showing that ClogP and pK_a _are important descriptors. Other authors have developed increasingly sophisticated models, introducing more complicated Quantitative Structure-Property Relationship (QSPR) methods and descriptors [13-15].

In this study, our aim is to use an *in silico *approach to predict the possible targets that may be relevant for phospholipidosis. By predicting the targets for a database of phospholipidosis-inducing compounds, we can rank targets by their potential to cause phospholipidosis and compare them to targets previously suggested.

The study of off-target interactions, known as secondary pharmacology, is now recognised as crucial to the understanding of both drug action and toxicology. In favourable cases, one drug may modulate plural disease-relevant targets, a property known as polypharmacology. More commonly, off-target interactions present the risk of side-effects, as is the case with phospholipidosis. Given the prevalence, expense, and risk to patients associated with unforeseen side-effects related to drug-target interactions, studies in this area have particular relevance to the pharmaceutical industry.

This study uses a methodology more complex than many seen in cheminformatics. Our objective is not simply to appeal to the similar property principle. A prediction based on that would run something like this: molecule B is similar to molecule A, which induces phospholipidosis, hence we predict that molecule B induces phospholipidosis too. Here, by way of contrast, we are interested in teasing out a mechanistic understanding much richer than can be obtained by similarity searching or QSPR. Thus, our interest is in predicting compound-target associations that will allow us to understand how phospholipidosis is induced and in suggesting and informing experimental approaches directed towards gaining a deeper mechanistic understanding.

## Materials and methods

The ChEMBL database [16] was mined for compounds and their related protein targets. A number of rules were used to filter the dataset. Only compounds which had an associated structure were selected. If the target description included the word "enzyme", "cytosolic", "receptor", "agonist" or "ion channel" and the bioactivity record of the compound contained an IC50, K_i _or K_d _< 500 μM or had an activity > 50% binding affinity, then it was selected. Of course, we recognise that differences between these measures may sometimes be significant; for instance, K_i _and K_d _are not strictly equivalent quantities. This selection process produced a dataset which consisted of compounds and their corresponding targets, where a compound may be related to more than one target. A relatively high IC50, K_i _or K_d _threshold was used as the aim of the study is to look at off-target prediction and therefore potentially weak binding targets. This approach selected a total of 249358 compounds which are related to a total of 3493 different targets. A further stipulation was that for a target to be present in the dataset it must have at least 20 compounds associated with it. This reduced the total dataset to 241145 compounds with 1923 different targets. In other words, the procedure yields *N *(= 241145) molecules belonging to *M *(= 1923) classes.

In the following discussion, the molecules are represented by pattern vectors (descriptors) **x***_j _*of dimension *d *with *j *= 1, 2, ... *N*; *ω_α _*denotes the classes with α = 1, 2, ... , *M*. In this work the descriptors used for the molecules were circular fingerprints [17]. To build a predictive model, the Parzen-Rosenblatt Window method [18] was used as the basis of a multi-class classification algorithm. For each possible class *ω_α_*, we used the Parzen-Rosenblatt Window scheme to estimate the average similarity of the test molecule **x***_i _*to the training set molecules in that class (say) **x***_j _*∈ *ω_α _*with similarity being measured by the kernel function *K*(**x***_i_*, **x***_j_*) as

(1) $$S_{\alpha }^{i}=\frac{1}{N_{\alpha }}\;\underset{x_{j}\in \;\omega_{\alpha }}{\sum }K\left(x_{i},\;x_{j}\right),$$

where *N*_α _denotes the number of the training data instances belonging to class *ω_α _*and the kernel function is as defined below. We wish to rank the classes for each compound according to our best estimates of the class probabilities *p*(*ω_α _*|**x***_i_*), the probabilities of the molecule being associated with each specific protein target. From Bayes' theorem, we can relate *p*(*ω_α _*|**x***_i_*) to *p*(**x***_i _*|*ω_α_*), the class-condition probability density (mass) function for molecule **x***_i _*given that it comes from class *ω_α _*, as follows:

(2) $$p\left(\omega_{\alpha }|x_{i}\right)=\frac{p\left(\omega_{\alpha }\right)p\left(x_{i}|\omega_{\alpha }\right)}{p\left(x_{i}\right)}\;$$

Since, for a given molecule, *p*(**x***_i_*) takes a constant value for all classes, ranking the classes by *p*(*ω_α _*|**x***_i_*) is equivalent to ranking them according to the product *p*(*ω_α_*)×*p*(**x***_i _*|*ω_α_*). This is a convenient approach, since both *p*(*ω_α_*) and *p*(**x***_i _*|*ω_α_*) are relatively easy to estimate. We take *p*(*ω_α_*) to be equal to the proportion of training set molecules belonging to that class, given by *N_α_/N*. It is assumed that *p*(**x***_i _*|*ω_α_*) is directly proportional to *S^i^_α_*, the measure of average similarity, as described in equations (1) and (3)

(3) $$p\left(x_{i}|\omega_{\alpha }\right)=\frac{1}{N_{\alpha }}\;\underset{x_{j}\in \;\omega_{\alpha }}{\sum }K\left(x_{i},\;x_{j}\right).$$

As well as the top-ranked class, effectively a prediction of the primary pharmaceutical target of a drug, we are equally interested in lower ranked predictions corresponding to off-target interactions potentially causing side-effects. We choose the Gaussian kernel

(4) $$K\left(x_{i},x_{j}\right)=\frac{1}{{\left(h^{2}\sqrt{\pi }\right)}^{d}}exp\left(-\frac{{\left(x_{i}-x_{j}\right)}^{T}\left(x_{i}-x_{j}\right)}{2h^{2}}\right),$$

where (**x***_i _*- **x***_j_*)*^T^*(**x***_i _*- **x***_j_*) corresponds to the number of features in which **x***_i _*and **x***_j _*disagree, while *h *is the so-called smoothing factor. In the scenario where equal probabilities are calculated for two classes, *p*(*ω_α_*)×*p*(**x***_i _*|*ω_α_*) = *p*(*ω_α_*)×*p*(**x***_i _*|*ω_α_*), these classes are ranked arbitrarily.

The mined ChEMBL dataset was partitioned into ten randomly split training and validation partitions, the size of which was determined by 99% of each class being present in the training and 1% in the validation set. For classes with fewer than 100 instances, a single instance was present in the validation and the rest in the training set. This produces a training data set with 238086 compounds and a validation set of 3059 compounds for each of the ten partitions. The Parzen-Rosenblatt Window method was applied to each of the ten splits with the smoothing factor *h *being varied according to 2^-15^, 2^-13^, ... , 2^3^. We also carried out analogous calculations using the Naïve Bayes method, implemented as described in reference [19], allowing us to compare the results from these two techniques.

**Figure 1:**
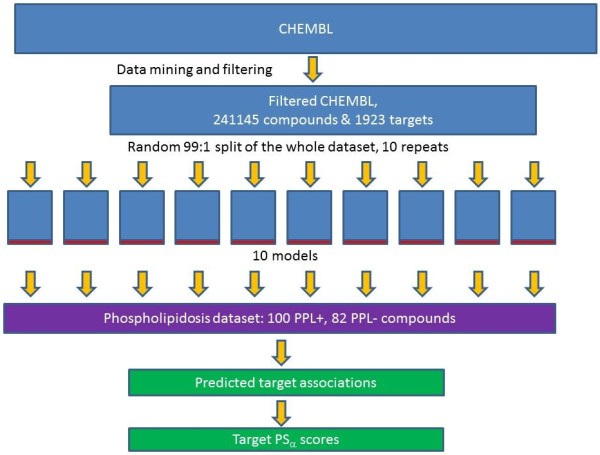
**Study methodology**. This Figure shows the overall methodology of mining ChEMBL, generating ten separate cross-validated models, applying these to the phospholipidosis dataset, and obtaining the *PS_α _*scores.

The ten different models produced on the ten different training partitions were then used to predict the targets of a phospholipidosis dataset with the Parzen-Rosenblatt Window method. The dataset consists of 182 compounds (100 are positive (PPL+) for phospholipidosis and 82 are negative (PPL-)) with a label indicating whether a compound is positive and induces phospholipidosis or is labelled negative and is experimentally confirmed to not induce it. We emphasise that all positives and negatives in our data are experimentally confirmed as such; there are no unverified assumed negatives. The data were primarily derived from Pelletier *et al. *[14], with a number of additional molecules taken from other literature sources such as [20], and are almost identical to the dataset we used in [15]. The full dataset is presented as Additional File 1. We note that an instance is a compound-target relation and not simply a compound, so another target association of a compound from the phospholipidosis dataset may appear in our training set. As we are interested in obtaining as comprehensive as possible a set of targets for these compounds, the other known compound-target relations were not removed from the training set. Our approach allows experimentally known associations of these 182 compounds with other targets, not directly relevant to phospholipidosis, to contribute to our predictions. From the targets predicted for each compound, the top 100 were used as this corresponds to approximately 5% of the total targets. As we are interested in off-targets, the order in which the targets were predicted for each compound is of limited interest here and hence a scoring system was designed to account for this. For the phospholipidosis dataset we have a label, *c_p_*, which represents whether a compound, **x***_i_*, is PPL+ (*c_p_*(**x***_i_*)=+1) or PPL- (*c_p_*(**x***_i_*)=-1). For each target, *ω_α_*, we calculate the phospholipidosis score *PS_α _*using equation (5):

(5) $$PS_{\alpha }=\overset{N}{\underset{i=1}{\sum }}C_{p}\left(x_{i}\right)\delta \left(\omega_{\alpha }\right)$$

where *δ(ω_α_) *= 1 if *ω_α _*is in the top 100 predictions or 0 otherwise, and *N *is the total number of compounds in the phospholipidosis dataset. The *PS_α _*score reported is a sum over the ten different models. A diagrammatic overview of our methodology is given in Figure 1.

## Results

The output recorded from the prediction of the validation compounds was the rank order of classes based on their estimated values of the product *p*(*ω_α_*)×*p*(**x***_i _*|*ω_α_*). The class with the highest probability was given a rank of 1, the second highest a rank of 2 and so on. To calculate the optimum smoothing parameter, the arithmetic mean of the rank of the actual class for the validation set was calculated. The smoothing factor *h *= 2^-3 ^produces the top average rank compared to the other smoothing factors tried and hence was used for the rest of the paper. Table 1 shows the arithmetic mean of the predicted ranks of actual experimentally known classes, calculated across the ten validation partitions with this smoothing parameter. Since this Table measures the prediction performance of our machine learning method, we excluded all data for validation compounds from the respective training sets. We also calculated predicted ranks in an exactly analogous way by 10-fold cross-validation using the Naïve Bayes method; these results are also shown in Table 1. The Parzen-Rosenblatt Window consistently assigns better ranks to the known targets, its predicted ranks being numerically smaller by a factor of 4.3. The calculated p-value of p = 2.889 × 10^-15 ^confirms that at the 5% significance level the mean of the average rank from the Parzen-Rosenblatt Window is statistically significantly smaller than the mean of the average rank from Naïve Bayes. Hence, we did not consider Naïve Bayes further and used only the Parzen-Rosenblatt Window in the phospholipidosis part of the study.

**Table 1:** Comparison of the Parzen-Rosenblatt Window and Naïve Bayes methods

**Partition No**.	PRW Rank	NB Rank
1	17.049	74.104
2	16.343	76.251
3	18.424	79.078
4	16.212	73.539
5	17.339	73.535
6	18.630	77.244
7	20.694	78.560
8	18.870	74.464
9	16.584	76.235
10	18.200	78.077
Average	17.835	76.109

Average ranks of known targets as predicted in the 10-fold cross-validation by the Parzen-Rosenblatt Window [18] and by a Naïve Bayes method [19]. The Parzen-Rosenblatt Window, using h = 2^-3^, consistently assigns better ranks to the known targets, its predicted ranks being numerically smaller by a factor of 4.3.

Table 2 shows the top 20 scoring targets and their phospholipidosis scores *PS_α_*. The *PS_α _*score is the total score for a target across all 182 compounds over the ten models derived from the ten different partitions of the ChEMBL dataset. A large number of the targets that score highly are CNS (central nervous system) type targets, such as the sodium-dependent serotonin transporters, dopamine receptors and serotonin receptors, which are often the primary pharmaceutical targets of CADs. The *PS_α _*scores for all 1923 targets are given in Additional File 2.

**Table 2:** Top 20 *PS_α_* scores for targets

Rank	Name	*PS_α_*
1	5-hydroxytryptamine receptor 2B (r)	444
2	5-hydroxytryptamine receptor 2C (r)	443
3	D(2) dopamine receptor (r)	436
4	5-hydroxytryptamine receptor 1A (r)	409
5	Potassium voltage-gated channel subfamily H member 2 (h)	406
6	Sodium-dependent serotonin transporter (r)	394
7 =	D(3) dopamine receptor (r)	385
7 =	D(3) dopamine receptor (h)	385
9	Muscarinic acetylcholine receptor M5 (r)	379
10	Alpha-1D adrenergic receptor (r)	376
11	Alpha-1A adrenergic receptor (r)	371
12	Alpha-1B adrenergic receptor (r)	369
13	5-hydroxytryptamine receptor 2A (r)	367
14 =	Sodium-dependent serotonin transporter (h)	357
14 =	5-hydroxytryptamine receptor 1B (r)	357
16 =	Transporter (r)	350
16 =	Muscarinic acetylcholine receptor M1 (r)	350
18	Sodium-dependent dopamine transporter (r)	349
19	Sigma 1-type opioid receptor (h)	348
20	Sodium channel protein type 2 subunit alpha (h)	347

List of the top 20 targets ranked by their *PS_α _*scores across all 182 compounds over the ten models derived from the ten different partitions of the ChEMBL dataset. A higher *PS_α _*score suggests that more phospholipidosis positive than negative compounds are associated with the target. A large number of the highly placed targets in our *PS_α _*rankings are the intended drug targets of CADs. Each of the top 20 targets comes from either human (h) or rat (r). Tied ranks are denoted by =.

## Discussion

The average ranks of the actual targets in the validation set in Table 1 show that the models are on average able to predict the correct target in the top 1%. This suggests that using high IC50, K_i _and K_d _values, which correspond to low activity, to select the dataset still allows for good predictive models and hence that it is possible to predict weak binding. If the cut-off is increased to the top 5% of targets, then an increase is seen from 96.1% of the actual targets being present amongst those predicted to 98.8%. It was therefore decided to use the top 5% of targets (actually 100/1923) for the phospholipidosis dataset prediction. Using this higher number allows for more of the off-targets to be selected; as the top predicted targets will often be the intended drug target of the cationic amphiphilic drug (CAD) or targets closely related to it.

None of the expected phospholipidosis-relevant targets appear in the top 20 ranked targets using the *PS_α _*score. The highest scoring target that had been previously suggested was lanosterol synthase (LSS), which is in a tie for rank 114. A large number of the highly placed targets in our *PS_α _*rankings are the intended drug targets of CADs, which can be used as antiarrhythmics, α-blockers and antipsychotics targeting ion channel transporters (such as sodium-dependent serotonin transporter) [21], as well as D2/D3 dopamine and serotonin receptors [10]. We also note that a number of the targets are within the same protein family and hence these fill a large number of the higher ranked positions.

Importantly for our work, consideration of known biochemical function allows us to link predicted targets to particular mechanisms of inducing phospholipidosis. Sawada *et al. *[11] previously suggested a number of genes relevant to each of their proposed mechanisms and Table 3 shows the ranks of some of the related targets according to their *PS_α _*scores. We also note that muscarinic acetylcholine receptors M1, M3 and M5 up-regulate phospholipase C, which if inhibited directly can lead to phospholipidosis. Therefore it could be expected that inhibition of the appropriate muscarinic acetylcholine receptors could lead to decreased production of phospholipase C and hence phospholipidosis by a more complex variant of mechanism 1. The M5 and M1 receptors appear amongst the top 20 ranked targets in 9^th ^and joint 16^th ^positions, respectively; M3 is ranked joint 32^nd^. Since they were not part of any of our original mechanistic hypotheses based on Sawada *et al*.'s work, however, we exclude the M1, M3 and M5 receptors from the discussion which follows.

**Table 3:** *PS_α_* scores and ranks for phospholipidosis-relevant targets

Mechanism	Target	Rank	*PS_α_*
1	Sphingomyelin phosphodiesterase (SMPD) (h)	225	55
	Lysosomal Phospholipase A1 (LYPLA1) (r)	163 =	90
	Phospholipase A2 (PLA2) (h)	152 =	97

3	Elongation of very long chain fatty acids protein 6 (ELOVL6) (h)	1203 =	-10
	Acyl-CoA desaturase (SCD) (m)	610 =	0

4	3-hydroxy-3-methylglutaryl-coenzyme A reductase (HMGCR) (h)	456 =	10
	Squalene monooxygenase (SQLE) (h)	437 =	14
	Lanosterol synthase (LSS) (h)	114 =	134

Table of the targets suggested by Sawada *et al. *[11] which are included in our model and their ranks based on the *PS_α _*score; tied ranks are denoted by =. The targets are grouped into their different mechanisms: 1) Inhibition of phospholipase activity; 2) Inhibition of lysosomal enzyme transport (not represented in this table); 3) Enhanced phospholipid biosynthesis; 4) Enhanced cholesterol biosynthesis. While Sawada *et al. *worked with human hepatoma HepG2 cells, [11] we also consider the corresponding genes in other species. Where homologous ChEMBL targets from two species were part of our model, for instance both human and rat versions of lanosterol synthase appeared, the higher scoring one is listed in this table; all its entries are from human (h), rat (r) or mouse (m).

Table 3 shows the ranked positions of the various targets predicted by Sawada *et al. *Sphingomyelin phosphodiesterase (SMPD) is responsible for the breakdown of sphingomyelin into phosphocholine and ceramide. Inhibition of SMPD would cause accumulation of the phospholipid sphingomyelin. A build-up of sphingomyelin is associated with Niemann-Pick disease which is often linked to phospholipidosis [22]. Lysosomal phospholipase A2 (LYPLA2) has previously been linked with phospholipidosis, however, due to the lack of data in ChEMBL it was not present in the model. Only two compounds have an associated binding affinity with this target and hence the target did not meet the requirement of having data for at least 20 compounds. LYPLA1 and phospholipase A2 (PLA2) were present in the model and produced *PS_α _*scores of 90 and 97, respectively. We expect that lysosomal phospholipase A2 would produce a similar score. Both of these targets act by breaking down phospholipids and hence are shown in Table 3 as being associated with mechanism 1. Since there are no relevant targets present in the original training data, it is not possible to comment on the likelihood of mechanism 2. However, it is clear that our model predicts that the induction of phospholipidosis *via *the mechanism 3 targets ELOVL6 or SCD is unlikely, as neither is predicted to interact with any of the 100 positive phospholipidosis-inducing compounds. For mechanism 4, out of the targets included in our model, lanosterol synthase produced the best result of those related to Sawada *et al*.'s mechanisms. Lanosterol synthase is involved in steroid biosynthesis, catalysing the cyclisation of (S)-2,3 oxidosqualene to lanosterol; hence it is associated with enhanced cholesterol biosynthesis (mechanism 4).

Since three targets for mechanism 1 and one for mechanism 4 score highly, our results suggest that a combination of mechanisms 1 and 4 is responsible for inducing phospholipidosis. Thus we find support, from an independent source of evidence and a quite different methodology, for two of the four mechanisms (1 & 4) which Sawada *et al. *proposed on the basis of their gene expression experiments. A lack of data for suitable targets meant that we could not test any targets for their mechanism 2, while our study suggests that their mechanism 3 does not occur *via *the targets ELOVL6 or SCD. Our method can only predict drug-protein associations and cannot predict whether phospholipidosis will occur *via *drug-phospholipid binding. Therefore it can only predict a mechanism which involves direct interaction with the protein.

**Figure 2:**
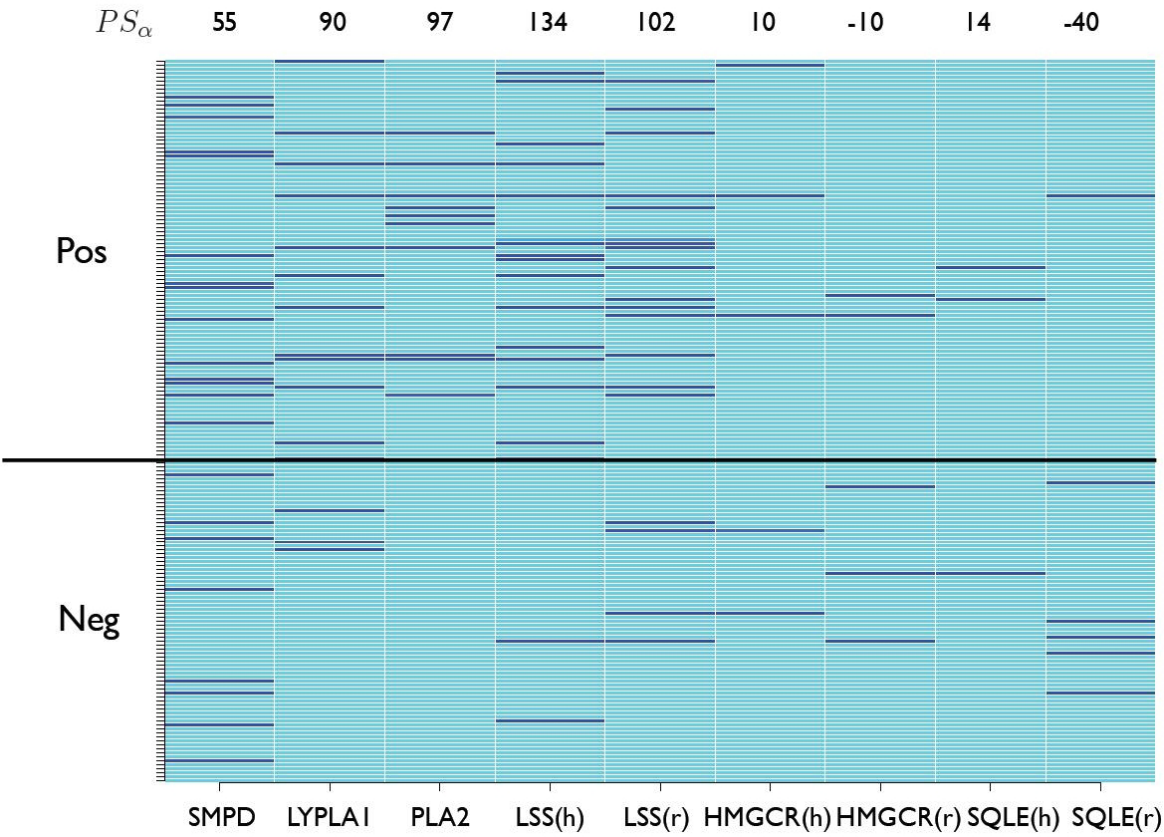
**Predicted interactions for phospholipidosis-relevant compounds and targets**. Figure showing the score (0 - 10) for nine different targets for each compound in the phospholipidosis dataset. The targets shown are the six Sawada targets for mechanisms 1 and 4 from Table 3, with both human (h) and rat (r) versions listed separately where data are available. A score of 10 means that the target was predicted for that compound in each of the ten runs of the Parzen-Rosenblatt method, using the same partitions as for Table 1, and corresponds to dark blue shading. The most prevalent light blue colour denotes a score of 0, indicating no predicted interaction in any model.

Figure 2 shows the scores for the compounds in our phospholipidosis data set, for each of the Sawada *et al. *targets. The targets for mechanism 3 have not been included as they do not score for any of the positive compounds. SMPD, LYPLA1, PLA2 and LSS show a large number of hits amongst the positive compounds (at the top of Figure 2) and many fewer hits for the negative compounds (at the bottom of Figure 2). The method cannot be 100% accurate and hence it may be expected that a few erroneous negative hits are present, however some of the negative hits for SMPD can be explained. Cloforex is labelled as negative in the dataset [23] but Ryrfeldt [24] suggested that it should be labelled as positive, and procaine is a CAD which does not induce phospholipidosis, perhaps due to its low logP.

It is also interesting to observe from Figure 2 that the compounds which are predicted to bind to SMPD are mostly different to those which are predicted to bind to LSS. A Pearson correlation coefficient of -0.847 was calculated between these two targets which suggests that there is some anti-correlation. A chi-squared test was used to assess the null hypothesis that the compounds' scores for LSS and SMPD are independent. The calculated p-value is 5.76 × 10^-5 ^and hence at the 5% significance level the null hypothesis is rejected. The lack of independence between the scores for these two targets, coupled with the observed anti-correlation, suggests that different compounds induce phospholipidosis *via *each of these two targets, which are associated with different mechanisms. We have also investigated the correlation between scores for other pairs of targets; the independence of scores between SMPD and LYPLA1 has an associated p-value of 0.507, and hence at the 5% level the null hypothesis that they are independent is not rejected. The Pearson correlation coefficient was calculated to be -0.247, suggesting that LYPLA1 and SMPD are anti-correlated.

**Figure 3:**
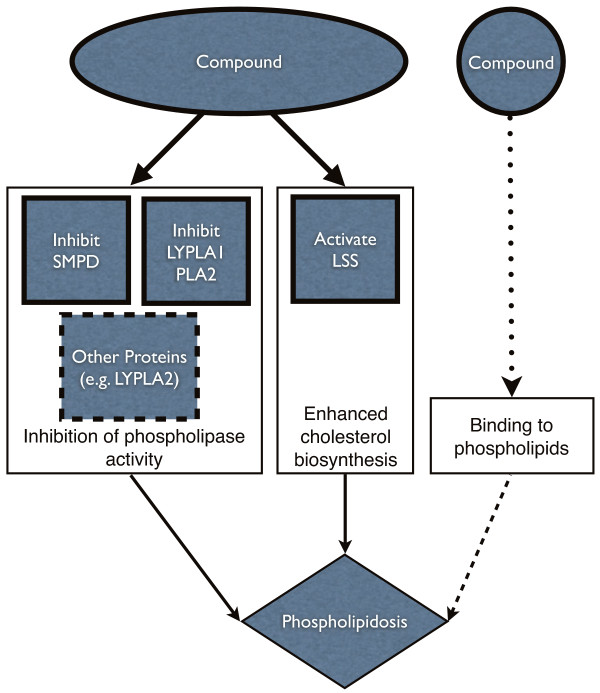
**Overview of the predicted mechanisms for phospholipidosis**. This Figure gives an overview of the predicted mechanisms for phospholipidosis. Solid lines indicate our predicted mechanisms of phospholipidosis induction. Dotted lines suggest other possible mechanisms or targets that were not present in our model.

Thus our results suggest that there is strong statistical evidence that no single target or even mechanism is responsible for phospholipidosis. We find that both inhibition of phospholipase activity and enhanced cholesterol biosynthesis are likely to be important mechanisms. Furthermore, this study provides evidence that sphingomyelin phosphodiesterase, phospholipase A2 and lysosomal phospholipase A1 are all likely targets for the induction of phospholipidosis by inhibition of phospholipase activity, while lanosterol synthase is expected to be associated with phospholipidosis occurring due to enhanced cholesterol biosynthesis. With these four targets, LSS, PLA2, LYPLA1, SMPD, and even the possible additional inclusion of muscarinic acetylcholine receptors M1, M3 and M5, we cannot account for all of the phospholipidosis-inducing compounds. Hence, we suspect that either more targets are involved or that compounds may induce phospholipidosis not only by interacting with protein targets, but also by binding to the lipid itself. An overview of the predicted mechanisms is presented in Figure 3.

## Conclusions

Using the Parzen-Rosenblatt Window method, predictive models of protein target associations were constructed based on compound structures. For our validation set, experimentally known targets were ranked (on average) in the top 1% of predicted targets. When applied to a dataset of phospholipidosis-inducing and non-inducing compounds, it was found that a number of targets may be linked to phospholipidosis. Sphingomyelin phosphodiesterase, lysosomal phospholipase A1, phospholipase A2 and lanosterol synthase all score highly according to our phospholipidosis score, *PS_α_*. It was shown that predicted activities against different targets are often uncorrelated or even anti-correlated. More simply put, different phospholipidosis-inducing compounds are predicted to interact with different putative phospholipidosis-relevant targets. This strongly suggests that different compounds induce phospholipidosis *via *different targets, and therefore also by different mechanisms. We note that, considering only the four different targets found to be significant here, there remain a number of PPL+ compounds for which a relevant target cannot be identified. This may indicate that further protein targets are mechanistically relevant, or that binding of the compound directly to the lipid is a possible mechanism.

## Competing interests

The authors declare that Robert Glen is a consultant with Unilever and Eisai and on the Board of Lhasa Ltd., and that there are no other conflicts of interest.

## Authors' contributions

RL wrote the software and carried out the calculations reported in this paper. JBOM, RL and FN jointly conceived the original idea and plan for this study. RL, HYM and RCG together developed the mathematical methodology used in this work. FN carried out preliminary computations upon which this work builds. RCG and JBOM provided regular supervisory input throughout the course of this work. RL wrote the original draft of the paper. All authors were closely involved in refining the manuscript and all read and approved the final version.

## Supplementary Material

Additional file 1 **The phospholipidosis dataset of 182 compounds**. We present the names, SMILES strings and phospholipidosis-inducing status of the 182 molecules; the file is in .xls format. Click here for file

Additional file 2 ***PS_α _*scores and ranks for all 1923 targets**. All 1923 targets ranked by their *PS_α _*scores across all 182 compounds over the ten models derived from the ten different partitions of the ChEMBL dataset. A higher *PS_α _*score suggests that more phospholipidosis positive than negative compounds are associated with the target; the numbers of positive and negative compound associations contributing to each target's *PS_α _*score are also shown in this Table, which is in .xls format. Click here for file
